# Draft Genome Sequence of *Rhodotorula mucilaginosa*, an Emergent Opportunistic Pathogen

**DOI:** 10.1128/genomeA.00201-15

**Published:** 2015-04-09

**Authors:** Massimo Deligios, Cristina Fraumene, Marcello Abbondio, Ilaria Mannazzu, Alessandro Tanca, Maria Filippa Addis, Sergio Uzzau

**Affiliations:** a Dipartimento di Scienze Biomediche, Università di Sassari, Sassari, Italy; b Porto Conte Ricerche, Parco Scientifico e Tecnologico della Sardegna, Alghero, Italy; c Dipartimento di Agraria, Università di Sassari, Sassari, Italy

## Abstract

*Rhodotorula mucilaginosa*, a yeast with valuable biotechnological features, has also been recorded as an emergent opportunistic pathogen that might cause disease in both immunocompetent and immunocompromised individuals. Here, we report the draft genome sequence of *R. mucilaginosa* strain C2.5t1, which was isolated from cacao seeds in Cameroon.

## GENOME ANNOUNCEMENT

*Rhodotorula* species are ubiquitous saprophytic yeasts that can be recovered from many environmental sources, including humans, animals, and a large variety of foods and beverages (1). Among these species, *Rhodotorula mucilaginosa* has been receiving increasing attention because it can be isolated not only from naturally fermented milk (2) and other food matrices (3, 4), but also from different and extreme ecosystems, including the complex core gut microbiota of carnivore wild fish (5), marine shores, glacial core cold environments (6), and hydrocarbon-contaminated soil (7).

In keeping with its capability to survive and grow in many unfavorable conditions, *R. mucilaginosa* possesses valuable biotechnological features, including copper biotransformation (8), the production of biosurfactants (9), and high yields of unsaturated fatty acids (6) and carotenoids (10). Moreover, yeasts ascribed to this species have a potential use in the biocontrol of postharvest fungal spoilage on fruit (11, 12).

Indeed, *R. mucilaginosa* also represents an emergent opportunistic pathogen for humans and animals, causing skin infections, otitis, lung infections, endocarditis, fungemia, and keratitis in both immunocompetent and immunocompromised individuals (1, 13–18).

Altogether, this information indicates that knowledge of the genomic potential of *R. mucilaginosa* should be extended to the exploration of the cellular physiology and biochemical and biotechnological potentials of this peculiar yeast species to facilitate research for both human and animal health and bioindustry.

*R. mucilaginosa* strain C2.5t1 was isolated from cacao seeds (*Theobroma cacao* L) in Cameroon. This isolate was previously assigned as *Rhodotorula glutinis* and fully characterized for its capability to produce high yields of biomass and carotenoids when grown in glycerol-containing media (19, 20). A DNA library was prepared using Nextera XT and sequenced using the Illumina HiScanSQ DNA sequencing platform. Sequence data generated about 57 million 2 × 93 bp paired reads that were assembled using Velvet v1/2/10 in 1,034 scaffolds containing 1,254 contigs longer than 500 bp (G+C content of 60.5%). Strain C2.5t1 assignment was confirmed by 18S rRNA gene identity of 100% to *R. mucilaginosa* strain MAFF237983 (accession no. AB042787) and identity of 99% to *R. glutinis* (accession no. HQ420261). A total of 6,413 protein-coding genes were automatically predicted using the Web server Augustus (http://bioinf.uni-greifswald.de/webaugustus) trained using *Ustilago maydis* parameters. Among these genes, 65% were assigned to protein functions using an identity ≥50% to a database containing *Sporidiobolales* (11,439 entries). As expected, among all predicted genes and protein functions, the genome of strain C2.5t1 showed a number of genes that might account for the ability of *R. mucilaginosa* to exert biotechnological features, including copper binding and biotransformation and pigment biosynthesis.

### Nucleotide sequence accession numbers.

The genome sequence of *R. mucilaginosa* strain C2.5t1 has been deposited at DDBJ/EMBL/GenBank under the accession no. JWTJ00000000. The version described in this paper is version JWTJ01000000.
